# Partial sequence identity in a 25-nucleotide long element is sufficient for transcriptional adaptation in the *Caenorhabditis elegans act-5*/*act-3* model

**DOI:** 10.1371/journal.pgen.1010806

**Published:** 2023-06-29

**Authors:** Jordan M. Welker, Vahan Serobyan, Elhamalsadat Zaker Esfahani, Didier Y. R. Stainier

**Affiliations:** Max Planck Institute for Heart and Lung Research, Department of Developmental Genetics, Bad Nauheim, Germany; University of Miami, UNITED STATES

## Abstract

Genetic robustness can be achieved via several mechanisms including transcriptional adaptation (TA), a sequence similarity-driven process whereby mutant mRNA degradation products modulate, directly or indirectly, the expression of so-called adapting genes. To identify the sequences required for this process, we utilized a transgenic approach in *Caenorhabditis elegans*, combining an overexpression construct for a mutant gene (*act-5*) and a fluorescent reporter for the corresponding adapting gene (*act-3)*. Analyzing a series of modifications for each construct, we identified, in the 5’ regulatory region of the *act-3* locus, a 25-base pair (bp) element which exhibits 60% identity with a sequence in the *act-5* mRNA and which, in the context of a minimal promoter, is sufficient to induce ectopic expression of the fluorescent reporter. The 25 nucleotide (nt) element in the *act-5* mRNA lies between the premature termination codon (PTC) and the next exon/exon junction, suggesting the importance of this region of the mutant mRNA for TA. Additionally, we found that single-stranded RNA injections of this 25 nt element from *act-5* into the intestine of wild-type larvae led to higher levels of adapting gene (*act-3)* mRNA. Different models have been proposed to underlie the modulation of gene expression during TA including chromatin remodeling, the inhibition of antisense RNAs, the release of transcriptional pausing, and the suppression of premature transcription termination, and our data clearly show the importance of the regulatory region of the adapting gene in this particular *act-5*/*act-3* TA model. Our findings also suggest that RNA fragments can modulate the expression of loci exhibiting limited sequence similarity, possibly a critical observation when designing RNA based therapies.

## Author summary

Transcriptional adaptation (TA) is a recently identified process that modulates the transcriptome of a cell in response to a mutation in a gene, independent of the mutation’s effect on the encoded protein. Depending on the identity of the gene(s) whose expression is modulated, TA can lead to functional compensation or a worse phenotype. TA requires mutant mRNA degradation, and the current hypothesis is that the resulting RNA fragments, or their derivatives, modulate the expression of the so-called adapting genes. A major unresolved question about TA is how the mutant and adapting gene(s) are linked, and it has been proposed that sequence similarity plays an important role. Here, we link a short DNA sequence in the regulatory region of an adapting gene (*act-3*) to a corresponding sequence in the mutant gene (*act-5*), and also show that small RNAs containing this *act-5* sequence element can lead to higher levels of adapting gene (*act-3)* mRNA. Because the sequence similarity between these two elements is lower than expected, these results are significant as they suggest that the number of potential adapting genes for a given mutant gene is currently underestimated.

## Introduction

Under the umbrella of various mechanisms contributing to genetic robustness, or the ability of a living cell or organism to maintain homeostasis in the presence of mutations, transcriptional adaptation (TA) is of particular interest because it regulates gene expression in response to mutant mRNA degradation and not protein feedback loops [1,2]. According to the current model of TA, in the presence of mRNA destabilizing lesions, mRNA degradation products, or their derivatives, translocate from the cytosol to the nucleus where they modulate the mRNA levels of the adapting gene(s). Notably, full locus deletion alleles do not exhibit TA [1,2], indicating that mutant mRNA molecules are required. As such, TA can lead to functional compensation [3–7] or to more severe phenotypes [8] depending on the gene(s) whose expression becomes modulated by the mutant mRNA degradation fragments and/or their derivatives. TA was first described in zebrafish, and offered as an explanation for the differences in phenotypes between knockdown and knockout animals [3]. Subsequently, TA was also reported in mouse cells in culture [1], in *Caenorhabditis elegans* [4,9], and in the green alga *Chlamydomonas reinhardtii* [10], suggesting that it is a widespread phenomenon. A major unresolved question about TA concerns the identity of the modulated genes, and initial observations suggest that sequence similarity plays an important role in their selection [1–4]. Therefore, identifying which sequences in the mutant mRNA are used to select adapting genes and also which sequences in the locus of the adapting genes are important for their modulation will further improve our understanding of the mechanisms underlying TA.

In order to address these questions we used a transgenic approach, which facilitates the identification of sequence elements necessary and sufficient for the TA response, especially when working with essential genes. Working in *C*. *elegans*, we have previously reported the ectopic expression of a fluorescent extrachromosomal reporter for *act-3* (the adapting gene) in in the intestine of *act-5* mutants that display mutant mRNA degradation (i.e., *act-5* mutants containing a premature termination codon (PTC) in exon 1) [4]. In a follow-up study, we reported that expression of an *act-5(ptc)* transgene (*eft-3p*:*act-5(ptc)*) leads to increased *act-3* mRNA levels as well as the *de novo* expression of an *act-3p*:*rfp* reporter transgene in a tissue where the *act-5(ptc)* transgene is expressed, namely the uterus [11]. Here, we use two extrachromosomal transgenes, a TA ‘driver’ (i.e., *eft-3p*:*act-5(ptc)*) and a TA ‘reporter’ (i.e., *act-3p*:*rfp*), to identify sequences on both the mutant transcript and the adapting gene locus that are necessary or sufficient for TA. After testing a series of truncations and rearrangements for each transgene, we identified, in the 5’ regulatory region of the *act-3* locus, a 25-base pair (bp) element that exhibits 60% identity with a sequence in the *act-5* mRNA and is sufficient for the TA response. We also tested the element identified in the mutant gene (*act-5*) by injecting small single-stranded RNAs (ssRNAs) and quantifying the adapting gene (*act-3*) mRNA levels.

## Results

### Ectopic uterine RFP expression as a proxy for the transcriptional adaptation response

**Fig 1:**
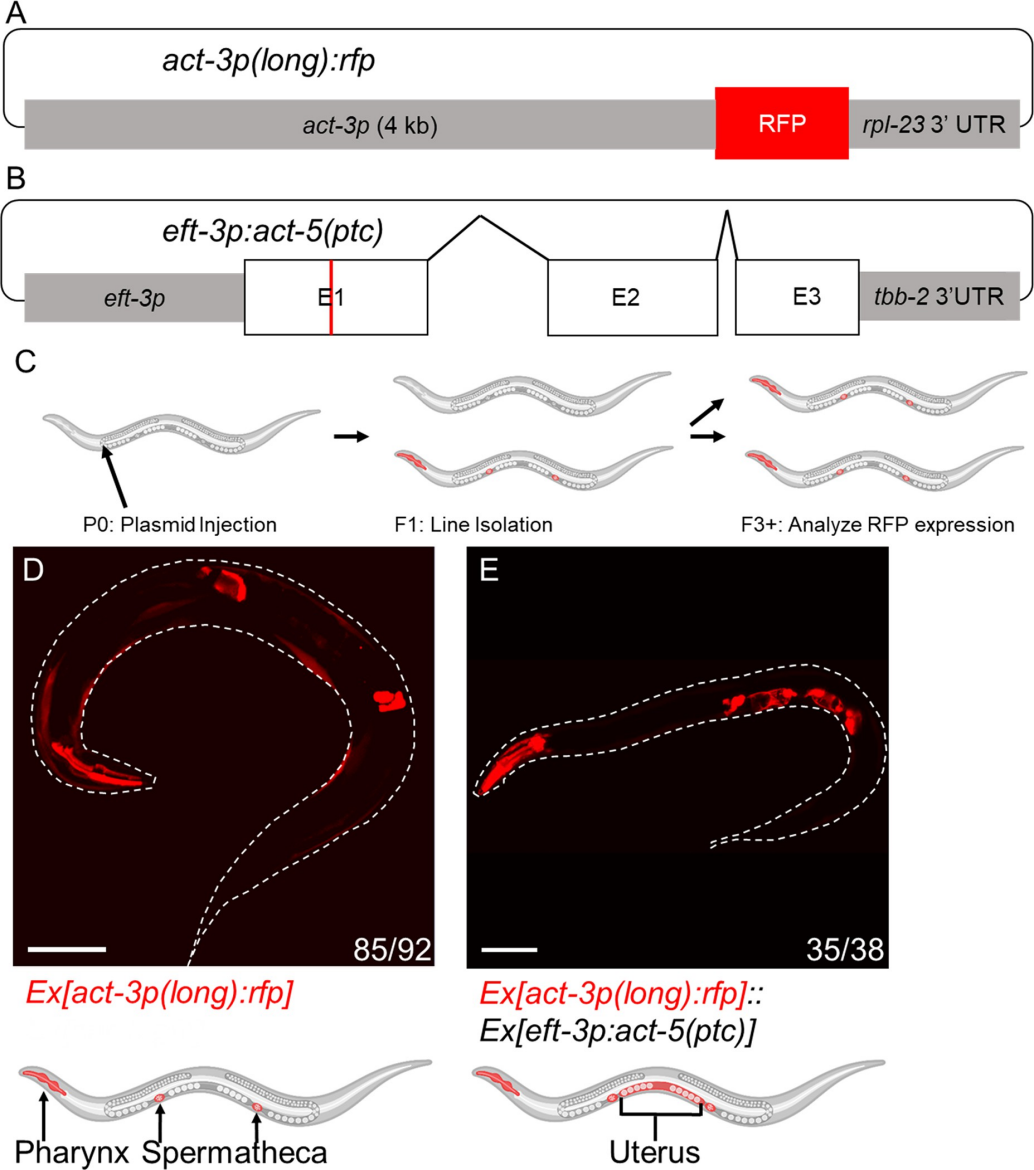
Ectopic uterine RFP expression as a proxy for the transcriptional adaptation response. (A) Diagram of the *[act-3p(long)*:*rfp]* reporter construct for the adapting gene (*act-3*), i.e., the TA ‘reporter’. A 4 kb sequence directly 5’ of the *act-3* translational start codon drives TurboRFP expression [4]. (B) Diagram of the overexpression construct for the mutant gene (*act-5*) [11], i.e., the TA ‘driver’; the ubiquitous *eft-3* promoter drives expression of the complete *act-5(ptc)* sequence including exons (E), and introns (bent lines); PTC marked with a red line. (C) Methodology for transgenic line generation: P0 WT animals were injected with a plasmid mix, fluorescent F1s were selected for line isolation, and expression patterns were analyzed starting in the third generation. (D) Representative image and cartoon of an animal containing only the *act-3p*:*rfp* reporter construct displaying reporter expression in the pharynx, body wall, and spermatheca [11]. (E) Representative image and cartoon of an animal containing both the *act-3p*:*rfp* reporter and *act-5(ptc)* overexpression constructs displaying reporter expression in the pharynx, body wall, spermatheca, and uterus. n = number of animals exhibiting the representative phenotype over the number of fluorescent animals screened. Cartoons were generated using Biorender.com (full license). Scale bars = 100 μm.

In order to identify the sequences in the *act-3* promoter and in the *act-5* mRNA that are necessary and/or sufficient for the TA response of *act-3*, we utilized two plasmids to generate transgenic animals. The first plasmid *[act-3p(long)*:*rfp]* uses a 4 kb promoter for the adapting gene (*act-3*) to drive TurboRFP expression (Fig 1A) [4]. The second plasmid *[eft-3p*:*act-5(ptc)]* uses a ubiquitous promoter to overexpress the mutant gene (*act-5(ptc)*) (Fig 1B) [11]. By analyzing transgenic animals containing one or both of these plasmids (Fig 1C), or their derivatives, we can identify the sequence requirements by comparing the RFP expression pattern between control and experimental animals. For this study, we define ‘control animals’ as animals injected with only the *[act-3p*:*rfp]* reporter (Fig 1D), and ‘experimental animals’ as animals injected with both the *[act-3p*:*rfp]* reporter and *[eft-3p*:*act-5(ptc)]* overexpression constructs (Fig 1E). For all transgene modification experiments, we compared the reporter expression pattern between control and experimental animals at the adult stage when the differences between uterus expression patterns are most evident (S1 Fig).

We first analyzed the *[act-3p(long)*:*rfp]* reporter and observed its expression in the pharynx, body wall, and spermatheca of transgenic animals (Figs 1D, S1A, and S1C), a pattern consistent with tissues known to express *act-3* [11–13]. We then asked whether there was a change in expression when the *[act-3p(long)*:*rfp]* reporter was co-injected with the *[eft-3p*:*act-5(ptc)]* overexpression construct, and observed an additional and strong RFP signal in the uterus of adult animals (Figs 1E, S1B, and S1D), as we previously reported [11]. This ectopic uterine expression phenotype is similar to the ectopic intestinal expression phenotype observed when the *[act-3p(long)*:*rfp]* reporter was exposed to the *dt2019(act-5(ptc))* mutation [4], in that we observe an expansion of the reporter expression into *act-5(ptc)* expressing tissues, indicating that we can use this system to visualize TA.

To further test whether the observed change in reporter expression was indeed due to TA, we co-injected the *act-3p*:*rfp* reporter and *act-5(ptc)* overexpression constructs into both the *ergo-1(gg100)* and *rrf-3(mg373)* mutant backgrounds [14]. We had previously reported that upregulation of the adapting gene (*act-3*) was blocked in both *act-5(ptc);ergo-1* and *act-5(ptc);rrf-3* double mutants [4]. Consistent with these data, we found that there was no uterine expression of the *[act-3p(long)*:*rfp]* reporter when co-injected with the *act-5(ptc)* overexpression construct in either of these mutant backgrounds (S2 Fig), further supporting the conjecture that in these experimental animals, TA drives the expression of RFP in the uterus.

### 25 base pairs in the *act-3* promoter are sufficient for the transcriptional adaptation response

**Fig 2:**
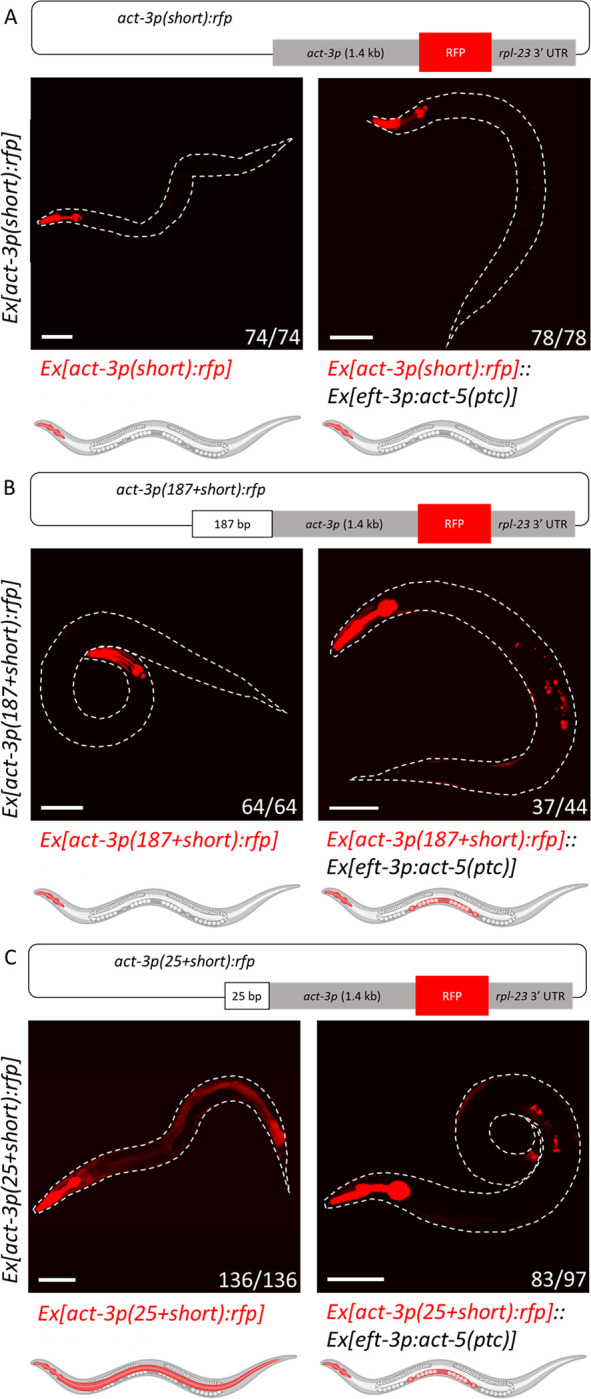
25 base pairs in the *act-3* promoter are sufficient for the transcriptional adaptation response. (A) Diagram of the *[act-3p(short)*:*rfp]* reporter construct. A 1.4 kb sequence directly 5’ of the *act-3* translational start codon drives TurboRFP expression. Representative image and cartoon of a control animal (left) displaying expression in only the pharynx, and representative image and cartoon of an animal (right) containing both the *act-3p*:*rfp* reporter and *act-5(ptc)* overexpression constructs displaying reporter expression in only the pharynx. (B) Diagram of the *[act-3p(187+short)*:*rfp]* reporter construct. 187 bp from the 5’ end of the *act-3p(long)* reporter construct were added to the 5’ end of the *[act-3p(short)*:*rfp]* reporter construct. Representative image and cartoon of a control animal (left) displaying expression in only the pharynx, and representative image and cartoon of an animal (right) containing both the *act-3p*:*rfp* reporter and *act-5(ptc)* overexpression constructs displaying reporter expression in the pharynx and uterus. (C) Diagram of the *[act-3p(25+short)*:*rfp]* reporter construct. Bases 163 to 187 (25 bp) from the *[act-3p(long)*:*rfp]* reporter construct were added to the 5’ end of the *[act-3p(short)*:*rfp]* reporter construct. Representative image and cartoon of a control animal (left) displaying expression in the pharynx and intestine, and representative image and cartoon of an animal (right) containing both the *act-3p*:*rfp* reporter and *act-5(ptc)* overexpression constructs displaying reporter expression in the pharynx and uterus. Worms outlined with a white dotted line; n = number of animals exhibiting the representative phenotype over the number of fluorescent animals screened. Cartoons were generated using Biorender.com (full license). Scale bars = 100 μm.

In order to determine which sequences in the *act-3* promoter are sufficient for TA, we first needed to identify a minimal promoter-driven reporter that exhibits no change in expression when co-injected with the *act-5(ptc)* overexpression construct. As many promoters in *C*. *elegans* are in the range of 1 to 2 kb [15], we removed 2.6 kb from the 5’ end of the *[act-3p(long)*:*rfp]* reporter leaving 1.4 kb directly upstream of the *act-3* translational start codon (Fig 2A). With this *[act-3p(short)*:*rfp]* reporter, we observed RFP expression only in the pharynx in both the control and experimental animals (i.e., no difference in RFP expression) (Fig 2A), suggesting that a sequence from the 2.6 kb region that was removed from the *[act-3p(long)*:*rfp]* reporter is involved in TA.

Next, we added sequences from this 2.6 kb region to the 5’ end of the *[act-3p(short)*:*rfp]* reporter to test for sufficiency, as well as tested internal deletions in the long promoter to test for necessity. For example, when we deleted 1.1 kb from the middle of the 2.6 kb region *[act-3p(long-1*.*1kb)*:*rfp]* (see methods), we observed ectopic reporter expression in the uterus when it was co-injected with the *act-5(ptc)* overexpression construct (S3 Fig), suggesting that this element is not necessary for TA. However, when a 187 bp sequence from the distal end of the long promoter was added to the 5’ end of the *[act-3p(short)*:*rfp]* reporter (*[act-3p(187+short)*:*rfp]*), we observed ectopic reporter expression in the uterus when it was co-injected with the *act-5(ptc)* overexpression construct (Fig 2B), suggesting that this 187 bp element is sufficient for TA in the context of the short/minimal promoter.

We note that the long promoter overlaps with the neighboring gene *act-2* (S4A Fig) [16]. Therefore, to determine which sub-sequence was sufficient for TA, the 187 bp was split into two parts (see methods). The first part consists of 162 bp of mostly exonic sequence; the second part consists of 25 bp of intronic sequence (S4B Fig). We found that when the 162 bp element was added onto the 5’ end of the short promoter reporter *[act-3p(162+short)*:*rfp]* and this construct was coinjected with the *act-5(ptc)* overexpression construct, RFP expression from the reporter was observed only in the pharynx (S4C Fig), suggesting that this 162 bp element is not sufficient for TA.

However, when the 25 bp element was added to the 5’ end of the short promoter reporter *[act-3p(25+short)*:*rfp]*, we first observed ectopic reporter expression in the intestine of the control animals in addition to expression in the pharynx (Fig 2C left panel), suggesting that in this particular location and in the absence of *act-5(ptc)* expression, this 25 bp element can act as an enhancer sequence. Additionally, when this *[act-3p(25+short)*:*rfp]* reporter was coinjected with the *act-5(ptc)* overexpression construct, we observed ectopic reporter expression in the uterus (Fig 2C right panel), suggesting that this 25 bp element is sufficient for TA in the context of the short/minimal promoter. In these experimental animals, intestinal expression was lost (Fig 2C right panel), suggesting that the mechanism of action of the enhancer effect (i.e., intestinal expression (Fig 2C left panel)) and TA are mutually exclusive, as has been reported for other transcription regulatory processes [17]. Notably, adding 10, 13, or 16 bases of this 25 bp element to the 5’ end of the short/minimal promoter was not sufficient to induce TA, indicating that the minimal length of the sufficient sequence is between 17 and 25 bp (S1 Table).

### Deleting the 25 bp sequence in the *act-3* reporter leads to its ectopic expression in the uterus

**Fig 3:**
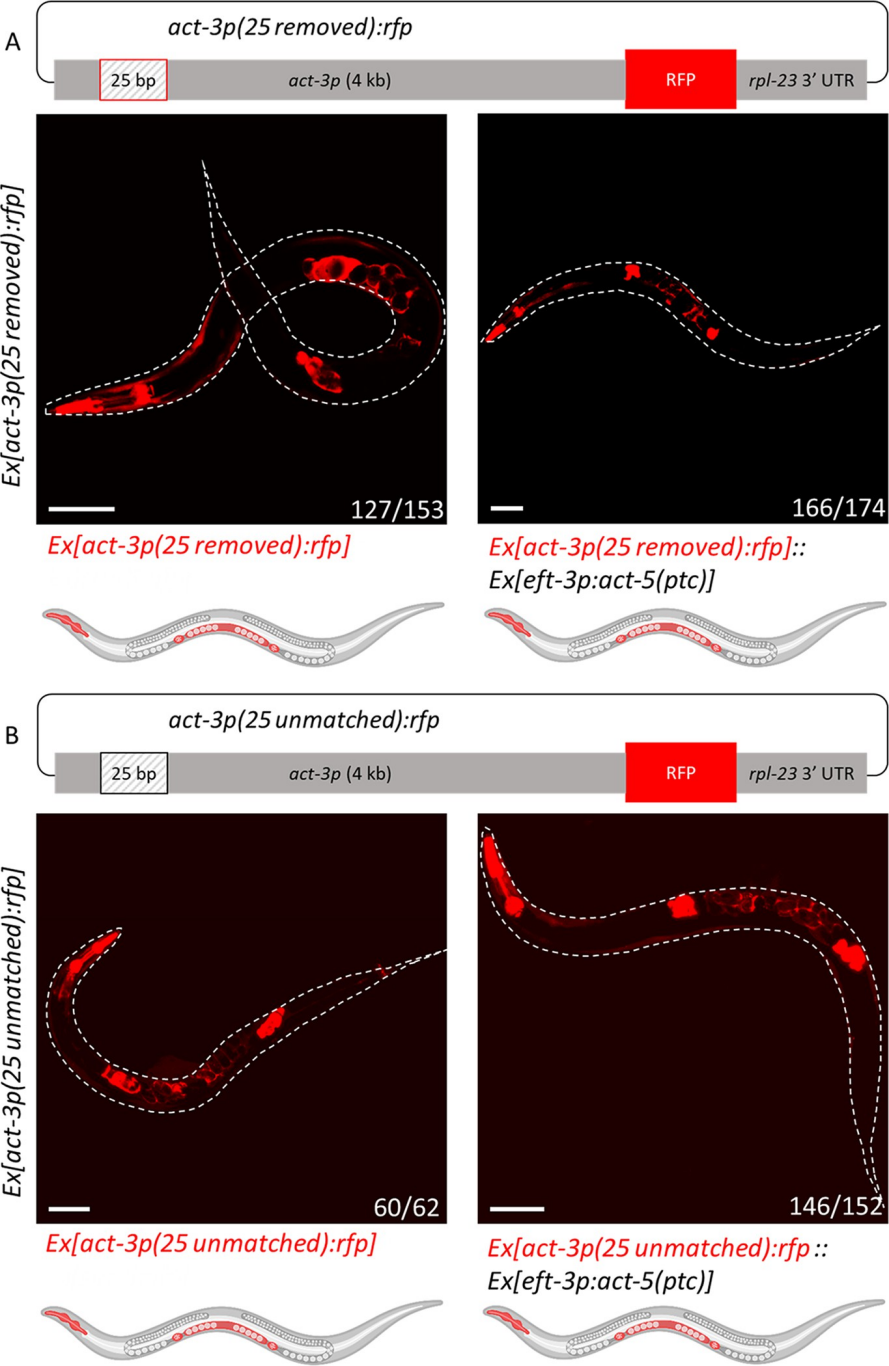
Deleting the 25 bp element in the *act-3* reporter leads to its ectopic expression in the uterus. (A) Diagram of the *[act-3p(25 removed)*:*rfp]* reporter construct. Bases 163 to 187 (25) bp were removed from the *[act-3p(long)*:*rfp]* reporter construct. The box outlined in red marks the location of the deleted element. Representative image and cartoon of a control animal (left) displaying expression in the pharynx, spermatheca, and uterus. Representative image and cartoon of an animal (right) containing both the *act-3p*:*rfp* reporter and *act-5(ptc)* overexpression constructs displaying reporter expression in the pharynx, spermatheca, and uterus. (B) Diagram of the *[act-3p(25 unmatched)*:*rfp]* reporter construct. Bases 163 to 187 (25) bp were altered to remove all sequence identity of the 25 bp in the *[act-3p(long)*:*rfp]* reporter construct. The box outlined in black marks the location of the unmatched element. Representative image and cartoon of a control animal (left) displaying reporter expression in the pharynx, spermatheca, and uterus. Representative image and cartoon of an animal (right) containing both the *act-3p*:*rfp* reporter and *act-5(ptc)* overexpression constructs displaying reporter expression in the pharynx, spermatheca, and uterus. Worms outlined with a white dotted line; n = number of animals exhibiting the representative phenotype over the number of fluorescent animals screened. Cartoons were generated using Biorender.com (full license). Scale bars = 100 μm.

To investigate whether this 25 bp element is necessary for TA, we deleted it from the *[act-3p(long)*:*rfp]* reporter (*[act-3p(25 removed)*:*rfp]*) and observed ectopic uterine expression in both the control and experimental (i.e., when coinjected with the *act-5(ptc)* overexpression construct) animals (Fig 3A). These data suggest that this 25 bp sequence is an important regulatory element of the *act-3* promoter, where it may be bound by a repressor. We made similar observations when we unmatched the 25 bp sequence *[act-3p(25 unmatched)*:*rfp]* (S1 Table), finding ectopic reporter expression in the uterus in both the control and experimental animals (Fig 3B), suggesting that the sequence of this element is critical for its function.

To investigate whether this 25 bp element plays a similar regulatory role in the endogenous locus, we deleted most of this element (leaving only one base behind to maintain the splice donor sequence in the neighboring gene, see methods), and observed that *act-3* was significantly upregulated in such mutants (S5A Fig). This result strengthens the suggestion that this 25 bp element is likely bound by a transcriptional repressor in its endogenous context, and that its removal, or alteration, leads to increased mRNA levels, independently of TA.

### Removing the 25-base pair sequence from the *act-5(ptc)* transgene diminishes the transcriptional adaptation response

**Fig 4:**
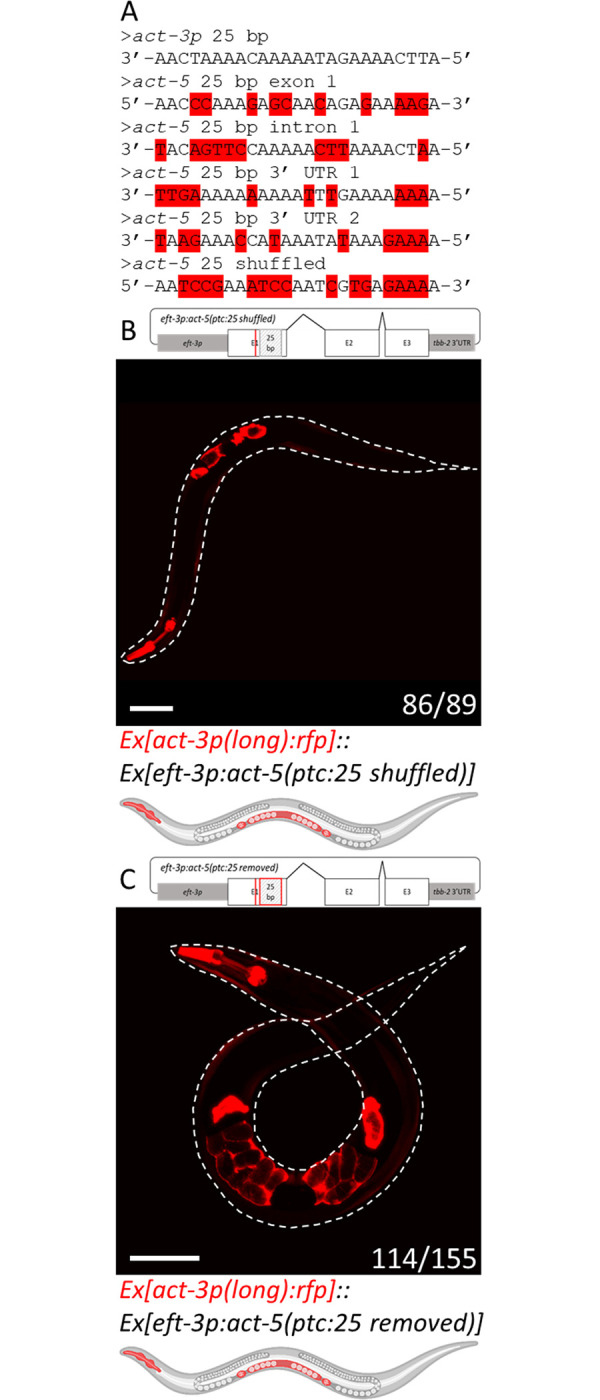
Removing the 25-base pair element from the *act-5(ptc)* transgene diminishes the transcriptional adaptation response. (A) Alignment of the *act-3p* 25 bp element to four *act-5* mRNA 25 bp sequences (60% identity), and to the *act-5* 25 bp shuffled sequence (40% identity), mismatched bases highlighted in red. (B) Diagram of the *[eft-3p*:*act-5(ptc*:*25 shuffled)]* construct with the 25 bp element shuffled (light grey box); representative image and cartoon of an animal containing both the *act-3p*:*rfp* reporter and *act-5(ptc)* overexpression constructs displaying reporter expression in the pharynx, body wall, spermatheca, and uterus. (C) Diagram of the *[eft-3p*:*act-5(ptc*:*25 removed)]* construct with the 25 bp element removed (red box); representative image and cartoon of an animal containing both the *act-3p*:*rfp* reporter and *act-5(ptc)* overexpression constructs displaying reporter expression in the pharynx, body wall, spermatheca, and uterus. Worms outlined with a white dotted line; n = number of animals exhibiting the representative phenotype over the number of fluorescent animals screened. Cartoons were generated using Biorender.com (full license). Scale bars = 100 μm.

To identify the *act-5* sequence that corresponds to the 25 bp element in the *act-3* promoter, we performed a sequence identity search (see methods). We identified four locations within the *act-5* sequence that are most similar to the 25 bp *act-3* sequence (60% identity) (Fig 4A); one is located in the first exon, another in the first intron, and the other two in the 3’ UTR. We excluded the intron element since it is not present in the mature RNA, and decided to focus on the 25 bp element located in the first exon of *act-5* as it contains the highest G/C content and thus would exhibit the greatest binding affinity between the two loci.

To determine whether this 25 bp element in *act-5* is necessary for TA in these transgenic conditions, we first shuffled its codons [18] to conserve the amino acid sequence of ACT-5 but reduce the identity as much as possible between the *act-5* and *act-3* DNA sequences, thereby reducing the identity from 60% to a final identity of 40% (Fig 4A). We found that in these experimental animals, co-injection of the *[eft-3p*:*act-5(ptc*:*25 shuffled)]* overexpression construct could induce uterine expression of the *[act-3p(long)*:*rfp]* reporter (Fig 4B), suggesting that this particular 25 bp element from *act-5* is not necessary for TA, or that 40% identity between the two sequences is sufficient. We then removed this 25 bp element from the *act-5(ptc)* plasmid and observed a significant reduction (*P* = 0.0124) in the percentage of experimental animals displaying ectopic uterine expression of the RFP reporter (Fig 4C): 73% of the *[act-3p(long)*:*rfp]*::*[eft-3p:act-5(ptc*:*25 removed)]* animals displayed ectopic reporter expression in the uterus compared with 92% in *[act-3p(long)*:*rfp]*::*[eft-3p:act-5(ptc)]* animals, and the other 27% looked like the control animals (Fig 1D), suggesting that the removal of this element from *act-5* affects the efficacy of the TA effect. These results suggest that other elements in the mutant mRNA may play a role in the TA response, or that in this experimental scenario, TA is induced indirectly by the *[eft-3p:act-5(ptc*:*25 removed)]* transgene via the triggering of an endogenous RNAi pathway [19] that leads to the degradation of the endogenous *act-5* mRNA.

### 25 nucleotide long single-stranded RNA injections lead to higher levels of *act-3* mRNA

**Fig 5:**
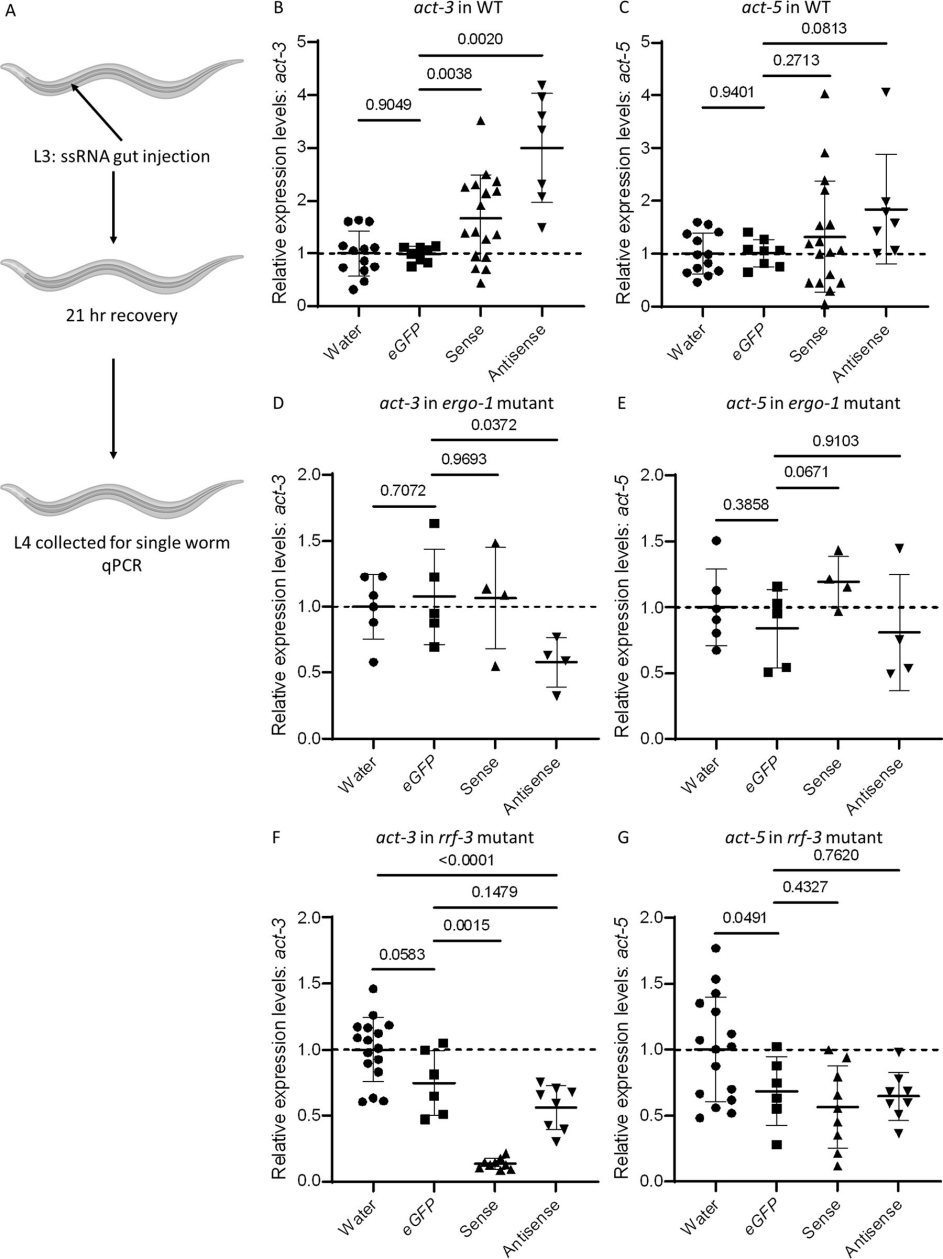
25 nucleotide long single-stranded RNA injections lead to higher levels of *act-3* mRNA. (A) Diagram of the ssRNA injection method. Sense or antisense ssRNA matching the *act-5* mRNA 25 nt element was injected into the gut of L3 stage animals. After a 21 hr recovery period, animals were collected for single worm RT-qPCR quantification of *act-3* and *act-5* mRNA levels relative to *cdc-42*. (B) Relative mRNA levels of *act-3* in WT animals injected with water, *eGFP* ssRNA, sense *act-5* ssRNA, and antisense *act-5* ssRNA. (C) Relative mRNA levels of *act-5* in WT animals injected with water, *eGFP* ssRNA, sense *act-5* ssRNA, and antisense *act-5* ssRNA. (D) Relative mRNA levels of *act-3* in *ergo-1* mutant animals injected with water, *eGFP* ssRNA, sense *act-5* ssRNA, and antisense *act-5* ssRNA. (E) Relative mRNA levels of *act-5* in *ergo-1* mutant animals injected with water, *eGFP* ssRNA, sense *act-5* ssRNA, and antisense *act-5* ssRNA. (F) Relative mRNA levels of *act-3* in *rrf-3* mutant animals injected with water, *eGFP* ssRNA, sense *act-5* ssRNA, and antisense *act-5* ssRNA. (G) Relative mRNA levels of *act-5* in *rrf-3* mutant animals injected with water, *eGFP* ssRNA, sense *act-5* ssRNA, and antisense *act-5* ssRNA. n ≥ 4 biologically independent samples. Data compared with water injected controls. Data are mean ± s.d., and a two-tailed Welch’s t-test was used to calculate *P* values. Cartoons were generated using Biorender.com (full license). *Ct* values are listed in S4 Table.

To determine whether 25 nt of RNA from *act-5* are sufficient to alter the mRNA levels of the endogenous adapting gene (*act-3*), we injected both sense and antisense single-stranded RNA (ssRNA) [20] into the gut of wild-type L3 larvae. After a 21-hour recovery period, worms were collected for single worm RT-qPCR [21] to assess the effects of *act-5* ssRNA injections on *act-3* mRNA levels; and we also assessed *act-5* mRNA levels (since the *act-5* ssRNAs match the *act-5* sequence exactly and they could have some RNA interfering activity) (Fig 5A). In order to control for the effects of the physical trauma of injection, and for any physiological alterations due to the presence of excess ssRNA, we injected water as well as a non-targeting 25 nt *eGFP* sequence. We observed no significant difference between water and *eGFP* ssRNA injections in WT animals in terms of *act-3* and *act-5* mRNA levels. (Fig 5B and 5C).

We found that injecting sense or antisense *act-5* ssRNA caused significant upregulation of *act-3* (Fig 5B), but had no effect on *act-5* mRNA levels (Fig 5C), when compared with controls, suggesting that the TA effect on the adapting gene (*act-3*) can be triggered by ssRNA matching the appropriate sequence from the mutant gene (*act-5*).

To assess whether the *act-5* ssRNAs are inducing *act-3* upregulation via TA, we performed injections into *ergo-1* mutants, which we previously reported fail to exhibit TA [4]. We found that, when compared with controls, injecting sense *act-5* ssRNA into *ergo-1* mutants caused no significant change in *act-3* mRNA levels, while injecting antisense *act-5* ssRNA caused a significant decrease in *act-3* mRNA levels (Fig 5D). In addition, there were no significant changes in *act-5* mRNA levels following *act-5* ssRNA injections into this mutant background (Fig 5E), suggesting that *ergo-1* is required for the increase in *act-3* mRNA levels after *act-5* ssRNA injections, and that the sense and antisense *act-5* ssRNA molecules may be used by different mechanisms to increase *act-3* mRNA levels in WT.

To further test whether the ssRNAs were inducing *act-3* upregulation via TA, we also performed injections into *rrf-3* mutants, which we previously reported also fail to exhibit TA [4]. We found that injecting *eGFP* ssRNA caused a non-significant decrease in *act-3* mRNA levels (Fig 5F), and a significant decrease in *act-5* mRNA levels (Fig 5G), suggesting that any ssRNA injections into this hypersensitive RNAi mutant [22] can lead to a global RNAi effect. We also found that injecting sense or antisense *act-5* ssRNA into *rrf-3* mutants caused a significant downregulation of both *act-3* and *act-5* mRNA levels (Fig 5F and 5G), when compared with water injected controls, possibly suggesting that *rrf-3* is required for the upregulation of *act-3* after *act-5* ssRNA injections.

To assess whether *act-5* ssRNA injections could induce upregulation of *act-3* when the 25 bp element was removed from its regulatory region, we performed injections into a deletion mutant. We observed no significant changes in *act-3* mRNA levels following *act-5* ssRNA injections into *act-2(knu112)* mutant animals when compared with controls (S5C Fig), suggesting that the effect induced by *act-5* ssRNA injections does not occur, or is masked (i.e., there is no additive effect/no significant change from the increased levels already present in these mutants), when the 25 bp element is removed from the *act-3* regulatory region. However, we observed significant upregulation of *act-5* in these mutant animals following antisense *act-5* ssRNA injection (S5D Fig), further suggesting that sense and antisense *act-5* ssRNA injections are modulating different regulatory mechanisms.

## Discussion

Transcriptional adaptation (TA) is a widespread cellular response to mRNA destabilizing lesions, including mutations, that is driven by mRNA degradation fragments, or their derivatives [1–7,11]. TA could lead to changes in gene expression via different mechanisms including chromatin remodeling [1,2,11], the inhibition of antisense RNAs [1], the release of transcriptional pausing [23], and the suppression of premature transcription termination [1–4,9]. Here, starting with a 4 kb piece of the *act-3* locus that includes the first exon and first intron as well as 2.8 kb of upstream sequence, we identified a 25 bp element 2.6 kb upstream of the transcriptional start site of the adapting gene (*act-3)* that is sufficient for TA in a transgenic setting. The corresponding 25 nt element in the mutant mRNA *(act-5)* lies between the PTC and the next exon/exon junction, suggesting the importance of this region of the mutant mRNA.

Generally, PTC containing transcripts are thought to initiate non-sense mediated mRNA decay (NMD) through stalling of the ribosome at the location of the PTC, and to lead to complete degradation of the aberrant mRNA molecule by the exosome and the exoribonuclease XRN1 [24]. Previous studies have shown that mRNA degradation via NMD can begin with endonucleolytic cleavage at the location of the PTC or slightly 3’ (i.e., downstream) of the PTC [25,26], and another study indicates that different locations of the PTC lead to different TA effects [9]. Therefore, although degradation is thought to produce primarily single bases [27–29], our observations that an important element for TA lies between the PTC and the next exon/exon junction and that ssRNAs matching a mutant locus can lead to higher levels of adapting gene (*act-3*) mRNA suggest that mRNA degradation may also produce small fragments unique to a given mutation. These fragments could arise due to their being protected by NMD machinery proteins, secondary structures in the mRNA itself, or by the stalling of XRN1 during degradation [30].

The approach we used to identify elements involved in TA in the *act-5*/*act-3* model was focused specifically on the 5’ regulatory region of *act-3* since we had previously found that it was sufficient for the TA response using transgenic reporters [4]. It is important to note that other elements in the *act-3* locus may also play a role. For example, a 25 bp sequence with 60% identity to the 25 bp *act-5* element is also present in the third intron of *act-3*, and we have not tested its function. However, *act-2* is not upregulated in *act-5(ptc)* mutants [4], suggesting that the 25 bp element in intron 2 of *act-2* we found to be sufficient for upregulation of the *act-3* reporter in the presence of the *act-5(ptc)* transgene does not play a similar role in the regulation of *act-2* expression.

We also note that removal of the 25 bp element from the *act-3* reporter induces its ectopic expression (suggesting that this element is bound by a transcriptional repressor), that TA results in the upregulation of the adapting gene (*act-3*) [4], and that a small RNA matching part of the mutant gene’s mRNA can lead to higher levels of adapting gene (*act-3*) mRNA. Altogether, these data lead us to suggest that in this TA model, small RNA(s) derived from the mutant gene’s (*act-5*) mRNA and bound by an RNA binding protein, translocate to the nucleus [31] and interact with the repressor element in the regulatory region of the adapting gene (*act-3*) enabling its activation. While the end result appears similar to what is observed during RNA activation [32,33], the underlying mechanisms might be quite different. Additionally, the limited sequence similarity between the 25 bp elements (60% identity) has implications for gene expression regulation on a broader scale, and we suggest that although TA was first observed as a functionally compensating mechanism [3,5–7], RNA-driven gene regulation may have broader effects due to the nonspecific ways small RNAs can recognize their targets [34,35].

## Methods

### *C*. *elegans* culture conditions and strains

All *C*. *elegans* strains were maintained on 6 cm plates with nematode growth medium agar and fed with a lawn of E. coli OP50 grown in 500 μl Luria broth [36]. All *C*. *elegans* strains used in this study are listed in Table 1. Cultures were maintained at 18–20°C. In addition, to minimize the potential for laboratory evolution of the traits, new cultures of the strains were revived annually from frozen stocks. All plates with fungal or bacterial contamination were excluded from the experiments.

**Table 1:** *C*. *elegans* resource table.

Reagent type	Designation	Source or reference	Additional information
Strain, strain background	N2	CGC, Bristol strain	wild type
Strain, strain background	YY168	CGC, Pavelec et al., 2009 [14]	*ergo-1(gg100)*
Strain, strain background	YY13	CGC, Pavelec et al., 2009 [14]	*rrf-3(mg373)*
Strain, strain background	DYSEx1002	Jiang et al., 2022 [11]	*Ex1000[act-3p*::*rfp]* referred to in text as *“Ex[act-3p(long)*::*rfp]”*
Strain, strain background	DYSEx1003	This study	*Ex1002[act-3p(long)*::*rfp];Ex1003[eft-3p*::*act-5(ptc)*::*tbb-2 3’UTR]*
Strain, strain background	DYSEx1004	This study	*Ex1004[act-3p(long)*::*rfp]; Ex1005[eft-3p*::*act-5(ptc)*::*tbb-2 3’UTR]; ergo-1(gg100)*
Strain, strain background	DYSEx1005	This study	*Ex1006[act-3p(long)*::*rfp]; Ex1007[eft-3p*::*act-5(ptc)*::*tbb-2 3’UTR]; rrf-3(mg373)*
Strain, strain background	DYSEx1006	This study	*Ex1008[act-3p(short)*::*rfp]*
Strain, strain background	DYSEx1007	This study	*Ex1009[act-3p(short)*::*rfp];Ex1010[eft-3p*::*act-5(ptc)*::*tbb-2 3’UTR]*
Strain, strain background	DYSEx1008	This study	*Ex1011[act-3p(long-187)*::*rfp]*
Strain, strain background	DYSEx1009	This study	*Ex1012[act-3p(long-187)*::*rfp];Ex1013[eft-3p*::*act-5(ptc)*::*tbb-2 3’UTR]*
Strain, strain background	DYSEx1010	This study	*Ex1014[act-3p(1*.*1 kb remove)*::*rfp];Ex1015[eft-3p*::*act-5(ptc)*::*tbb-2 3’UTR]*
Strain, strain background	DYSEx1011	This study	*Ex1016[act-3p(187+short)*::*rfp]*
Strain, strain background	DYSEx1012	This study	*Ex1017[act-3p(187+short)*::*rfp];Ex1018[eft-3p*::*act-5(ptc)*::*tbb-2 3’UTR]*
Strain, strain background	DYSEx1013	This study	*Ex1019[act-3p(162+short)*::*rfp]*
Strain, strain background	DYSEx1014	This study	*Ex1020[act-3p(162+short)*::*rfp];Ex1021[eft-3p*::*act-5(ptc)*::*tbb-2 3’UTR]*
Strain, strain background	DYSEx1015	This study	*Ex1022[act-3p(25+short)*::*rfp]*
Strain, strain background	DYSEx1016	This study	*Ex1023[act-3p(25+short)*::*rfp];Ex1024[eft-3p*::*act-5(ptc)*::*tbb-2 3’UTR]*
Strain, strain background	DYSEx1017	This study	*Ex1025[act-3p(25 removed)*::*rfp]*
Strain, strain background	DYSEx1018	This study	*Ex1026[act-3p(25 removed)*::*rfp];Ex1027[eft-3p*::*act-5(ptc)*::*tbb-2 3’UTR]*
Strain, strain background	DYSEx1019	This study	*Ex1028[act-3p(25 unmatched)*::*rfp]*
Strain, strain background	DYSEx1020	This study	*Ex1029[act-3p(25 unmatched)*::*rfp];Ex1030[eft-3p*::*act-5(ptc)*::*tbb-2 3’UTR]*
Strain, strain background	COP2474	This study	*act-2(knu112)* [24bp deletion in intron 2]
Strain, strain background	COP2475	This study	*act-2(knu113)* [24bp deletion in intron 2]
Strain, strain background	DYSEx1021	This study	*Ex1031[act-3p(long)*::*rfp];Ex1032[eft-3p*::*act-5(ptc; 25 shuffled)*::*tbb-2 3’UTR]*
Strain, strain background	DYSEx1022	This study	*Ex1033[act-3p*::*rfp long];Ex1034[eft-3p*::*act-5(ptc; 25 removed)*::*tbb-2 3’UTR]*

### *C*. *elegans* transgenic and mutant line generation

Injections for generation of RFP reporter lines were performed as previously described [37,38], with the following modifications. Plasmids were purified twice using the FastGene Plasmid Mini Kits (FG-90402; Nippon Genetics) and injected at a final concentration of 100 ng/μl. *[act-3p*:*rfp]* and *[eft-3p*::*act-5(ptc)]* plasmids were injected at equal molar ratios based on total sequence length, i.e., 60 ng/μl *[act-3p(long)*:*rfp]* and 40 ng/μl *[eft-3p*::*act-5(ptc)]*, or 50 ng/μl *[act-3p(short)*:*rfp]* and 50 ng/μl *[eft-3p*::*act-5(ptc)]*. A mixture of 90 ng/μl *[act-3p*:*rfp]* and 10 ng/μl *[sur-5*::*gfp]* plasmids was injected into each worm to generate control RFP only lines.

COP2474—*act-2(knu112)* and COP2475—*act-2(knu113)* mutant strains were generated by InVivo Biosystems using a Cas12a mediated single strand donor knock-in strategy. A unique CRISPR target in the *act-2* intron was selected to prevent off-target cutting at the *act-3* and *act-1* loci. The donor oligo utilized homology on both sides of the 24 bp sequence (genomic location *C*. *elegans* PRJNA13758:WBcel235:V:11077086:11077109) [16] so that the resulting mutation would retain the splice donor and remove the majority of the 25 bp element (genomic location *C*. *elegans* PRJNA13758:WBcel235:V:11077086:11077110) [16]. These two mutants appear identical based on sequencing their *act-2* locus. Genotyping primers are listed in S2 Table.

### *C*. *elegans* construct generation

The previously described *[eft-3p*::*act-5(ptc)]* transgene [11] was cloned into a smaller vector backbone (pCFJ1662, a gift from Erik Jorgensen (Addgene plasmid # 51482; http://n2t.net/addgene:51482; RRID:Addgene_51482)) between the minimos transposon arms using Gibson cloning [39]. The truncations, deletions, and rearrangements of the *[act-3p*:*rfp]* reporter and *[eft-3p*::*act-5(ptc)]* overexpression constructs were generated by site-directed mutagenesis.

The 1.1 kb deletion was designed to delete three lncRNAs present in the *act-3* promoter region. Initial experiments with a truncation series in the *dt2019* (endogenous *act-5(ptc)*) mutant background suggested that removal of 187 bp from the 5’ end of the *[act-3p(long)*:*rfp]* reporter affected the TA response (S3 Table).

To investigate whether the 187 bp sequence was necessary for TA, we deleted it from the *[act-3p(long)*:*rfp]* reporter (*[act-3p(long-187)*:*rfp]*) and observed ectopic reporter expression in the uterus of both the control and experimental animals (S1 Table), suggesting that this 187 bp sequence contains an important regulatory element of the *act-3* promoter. The 187 bp element was split into 162 bp of more conserved sequence (161 bp of *act-2* exon 2 sequence plus the first base of the splice donor), and 25 bp of less conserved sequence starting at the second base of the slice donor and including the rest of the 187 bp sequence. Further dissection of the 25 bp element (i.e., 10, 13, and 16 bp) added to the 5’ end of the *[act-3p(short)*:*rfp]* reporter) was designed in 3 bp increments starting from the middle of the sequence.

Sequences of the constructs are listed in S1 Table. Primers used for cloning are listed in S2 Table. The key plasmids used in this study have been deposited at Addgene.

### *C*. *elegans* screening

From the third generation onwards, plates of transgenic animals were scored on days five or six of their life cycle when gravid adults were present but before the plates began to starve. Only fluorescent adult animals were scored, and only if the entire pharynx appeared to be expressing RFP under a dissection microscope. Animals were categorized as pharynx only, pharynx plus uterus, or pharynx plus intestine. The ratio of fluorescent animals was taken as the number of animals displaying only pharynx RFP expression over the total number of screened animals at each generation. The significance of this ratio was calculated using Welsh’s T-test comparing each experimental condition to the appropriate control condition. Raw counts, fluorescent ratios, and *P* values are listed in S1 Table.

### *C*. *elegans* sequence comparison

The program A Plasmid Editor (ApE) [40] (version 2.0.70.0) was used to perform sequence identity searches for short sequence lengths. Using the identified 25 bp element from the *act-3* promoter as the query sequence, the “Find…” function within ApE was used to identify matching locations within the *act-5* locus (genomic location *C*. *elegans* PRJNA13758:WBcel235:III:13604322:13606215) [16]. Searches were performed sequentially allowing for one additional mismatch each round until a sequence within *act-5* was identified as matching the query sequence. Four sequences were identified with 10 mismatches (60% identity): one is located in the first exon (40% G/C content), another in the first intron (24% G/C content), and the other two in the 3’ UTR (8% and 16% G/C content). Comparing the 25 bp *act-3* promoter sequence to the *act-5* mRNA sequence identifies the same exonic sequence and the same two 3’ UTR sequences.

### ssRNA microinjections

ssRNA microinjections [20] were performed with the following modifications. ssRNAs were synthesized by Merck or Integrated DNA Technologies with 5’ phosphorylation and 3’ 2-O methylation for stability [41]. ssRNAs were resuspended to a final injection concentration of 100 μM in DNase/RNase free water, and around 80 pg was injected into the gut of L3 larvae. The injected larvae were allowed to recover for 21 hours and then collected for RNA isolation at the L4 stage (i.e., before eggs are present). Obviously sick or dying worms were excluded from these experiments. ssRNA sequences are listed in S2 Table.

### RT-qPCR analysis

RT-qPCR was performed using a CFX Connect Real-Time System (Biorad) or QuantStudio 7 Pro Real-Time PCR System (Applied Biosystems). Single *C*. *elegans* worms were collected for RNA isolation as previously described [21] with the following changes. Worms were collected into 2 μl of lysis buffer, cDNA synthesis was performed using the Maxima First Strand cDNA synthesis kit (Thermo Fisher) as half reactions using the entire 2 μl of RNA as template, cDNA was diluted to 14 μl with water, and 1 μl of cDNA was used for each qPCR reaction. For the analysis of *act-2(knu112)* and *act-2(knu113)* mutants (S5A and S5B Fig), pools of synchronized L1/L2 animals were collected and 1–2 μg of RNA was used for the reverse transcription reaction as previously described [1,4,11]. All reactions were performed in at least technical duplicates and the results represent biological replicates of n ≥ 4. *cdc-42* was used as the sole reference gene as it was found to be the only reliable one out of four tested (*cdc-42*, *Y45F10D*.*4*, *pmp-3*, and *tba-1*). Primer sequences used for the RT-qPCR experiments are listed in S2 Table. Fold changes were calculated using the 2−ΔΔCt method in groups of experiments performed at the same time to control for batch effects. All *Ct* values and analysis groups are listed in S4 Table.

### Microscopy

Fluorescence images of single *C*. *elegans* were acquired using a Zeiss LSM 700 confocal microscope (Plan-Apochromat 10X/0.45 objective lens). Group images of *C*. *elegans* were acquired using a Nikon SMZ25 dissection microscope (SHR Plan Apo 1x WD:60 objective lens) equipped with a Nikon Digital Sight DS-Ri1 camera. Worms were immobilized by mounting them in polystyrene microbeads [42]. Images were processed with ZEN software (black edition). All figures were prepared using Microsoft PowerPoint.

### Cartoons

Cartoons were generated using Biorender.com (full license).

### Statistics and reproducibility

Statistical analysis was performed using GraphPad Prism 8. Data are mean ± s.d., and a two-tailed Welch’s t-test was used to calculate *P* values. No statistical methods were used to predetermine sample size. The experiments were not randomized. The investigators were not blinded to allocation during experiments and outcome assessment. All experiments were performed at least twice unless otherwise noted. *P* values for all pairwise comparisons for ssRNA injections are listed in S5 Table. *P* < 0.05 was accepted as statistically significant.

## Supporting information

S1 Fig Ectopic uterine RFP expression is most evident at the adult stage. (A) Representative image of the midsection of an adult animal containing only the *act-3p*:*rfp* reporter construct displaying reporter expression in the spermatheca. Combined transmitted and RFP channels on the top; RFP only channel in the middle; cartoon on the bottom. (B) Representative image of the midsection of an adult animal containing both the *act-3p*:*rfp* reporter and *act-5(ptc)* overexpression constructs displaying reporter expression in the spermatheca and uterus. Combined transmitted and RFP channels on the top; RFP only channel in the middle; cartoon on the bottom. (C) Group image of seven representative adult animals containing only the *act-3p*:*rfp* reporter construct displaying reporter expression in the pharynx, body wall, and spermatheca; cartoon below. (D) Group image of six representative adult animals containing both the *act-3p*:*rfp* reporter and *act-5(ptc)* overexpression constructs displaying reporter expression in the pharynx, body wall, spermatheca, and uterus; cartoon below. Worms outlined with a white dotted line. Cartoons were generated using Biorender.com (full license). Scale bars = 100 μm.
(TIF)

S2 Fig The transcriptional adaptation response is blocked in *ergo-1* and *rrf-3* mutants. (A) Representative image and cartoon of an *ergo-1* mutant animal containing both the *act-3p*:*rfp* reporter and *act-5(ptc)* overexpression constructs displaying reporter expression in the pharynx and spermatheca. (B) Representative image and cartoon of an *rrf-3* mutant animal containing both the *act-3p*:*rfp* reporter and *act-5(ptc)* overexpression constructs displaying reporter expression in the pharynx and spermatheca. Worms outlined with a white dotted line; n = number of animals exhibiting the representative phenotype over the number of fluorescent animals screened. Cartoons were generated using Biorender.com (full license). Scale bars = 100 μm.
(TIF)

S3 Fig The 1.1 kb internal deletion does not affect the transcriptional adaptation response. Diagram of the *[act-3p(long-1*.*1kb)*:*rfp]* reporter construct. A 1.1 kb sequence was removed from the middle of the 2.6 kb region in the *[act-3p(long)*:*rfp]* construct. Representative image and cartoon of an animal containing both the *act-3p*:*rfp* reporter and *act-5(ptc)* overexpression constructs displaying reporter expression in the pharynx, spermatheca, and uterus. Worms outlined with a white dotted line; n = number of animals exhibiting the representative phenotype over the number of fluorescent animals screened. Cartoons were generated using Biorender.com (full license). Scale bar = 100 μm.
(TIF)

S4 Fig Diagram of the adapting locus and sequence locations. (A) Diagram of the *act-2/act-3* locus (PRJNA13758:WBcel235) [16]. Exons (E) are displayed as UTRs (grey boxes) and coding sequence (white boxes), and introns are represented by bent lines. *act-3p(long)*, *act-3p(short)*, 1.1 kb deletion, and 187 bp sequences are also displayed as grey boxes. Tick marks represent 500 bp. (B) Diagram of the 5’ end of the *[act-3p(long)*:*rfp]* reporter construct. The 5’ 187 bp element is located in the neighboring *act-2* locus. The 187 bp element consists of the 3’ 161 bp of *act-2* exon 2 (white box), and 26 bp of *act-2* intron 2 (bent line). This element was further tested as the 5’ 162 bp segment, and the 3’ 25 bp segment. Sequences are listed below. (C) Diagram of the *[act-3p(162+short)*:*rfp]* reporter construct. 162 bp from the 5’ end of the *[act-3p(long)*:*rfp]* reporter construct were added to the 5’ end of the *[act-3p(short)*:*rfp]* reporter construct. Representative image and cartoon of a control animal (left) displaying reporter expression in only the pharynx. Representative image and cartoon of an animal (right) containing both the *[act-3p(162+short)*:*rfp]* reporter and *act-5(ptc)* overexpression constructs displaying reporter expression in only the pharynx. Worms outlined with a white dotted line; n = number of animals exhibiting the representative phenotype over the number of fluorescent animals screened. Cartoons were generated using Biorender.com (full license). Scale bar = 100 μm.
(TIF)

S5 Fig Deleting 24 bp of the 25 bp element in the *act-3* regulatory region leads to ectopic *act-3* expression. (A) Relative mRNA levels of *act-3* in WT, *act-2(knu112)* mutant, and *act-2(knu113)* mutant animals. (B) Relative mRNA levels of *act-5* in WT, *act-2(knu112)* mutant, and *act-2(knu113)* mutant animals. n ≥ 5 biologically independent samples. Data compared with WT controls. (C) Relative mRNA levels of *act-3* in *act-2(knu112)* mutant animals injected with water, *eGFP* ssRNA, sense *act-5* ssRNA, and antisense *act-5* ssRNA. (D) Relative mRNA levels of *act-5* in *act-2(knu112)* mutant animals injected with water, *eGFP* ssRNA, sense *act-5* ssRNA, and antisense *act-5* ssRNA. n ≥ 6 biologically independent samples. Data compared with water injected controls. Data are mean ± s.d., and a two-tailed Welch’s t-test was used to calculate *P* values. *Ct* values are listed in S4 Table.
(TIF)

S1 Table Raw count data, and sequence information. (XLSX)

S2 Table Cloning and RT-qPCR oligos. (XLSX)

S3 Table Promoter lengths and TA outcome for pilot truncation series. (XLSX)

S4 Table RT-qPCR *Ct* values. (XLSX)

S5 Table *P* values for ssRNA injections. (XLSX)
