# Supplementary figures and images for: Partial sequence identity in a 25-nucleotide long element is sufficient for transcriptional adaptation in the Caenorhabditis elegans act-5/act-3 model
Source: PLoS Genet. 2023 Jun 29;19(6):e1010806. doi: 10.1371/journal.pgen.1010806 (PMC10310345; doi:10.1371/journal.pgen.1010806)

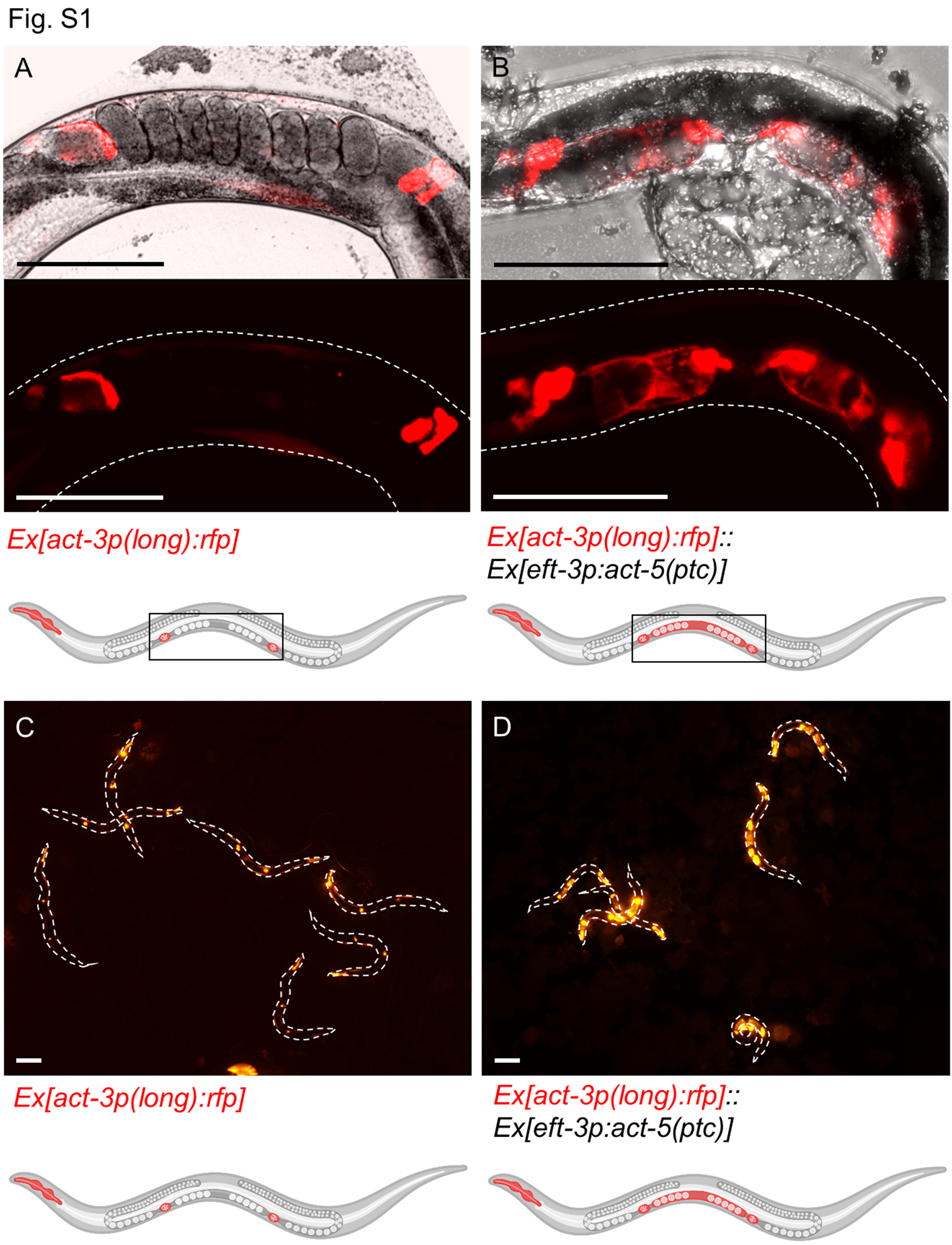

Supplement: S1 Fig — (A) Representative image of the midsection of an adult animal containing only the act-3p:rfp reporter construct displaying reporter expression in the spermatheca. Combined transmitted and RFP channels on the top; RFP only channel in the middle; cartoon on the bottom. (B) Representative image of the midsection of an adult animal containing both the act-3p:rfp reporter and act-5(ptc) overexpression constructs displaying reporter expression in the spermatheca and uterus. Combined transmitted and RFP channels on the top; RFP only channel in the middle; cartoon on the bottom. (C) Group image of seven representative adult animals containing only the act-3p:rfp reporter construct displaying reporter expression in the pharynx, body wall, and spermatheca; cartoon below. (D) Group image of six representative adult animals containing both the act-3p:rfp reporter and act-5(ptc) overexpression constructs displaying reporter expression in the pharynx, body wall, spermatheca, and uterus; cartoon below. Worms outlined with a white dotted line. Cartoons were generated using Biorender.com (full license). Scale bars = 100 μm. (TIF) [file pgen.1010806.s001.tif]

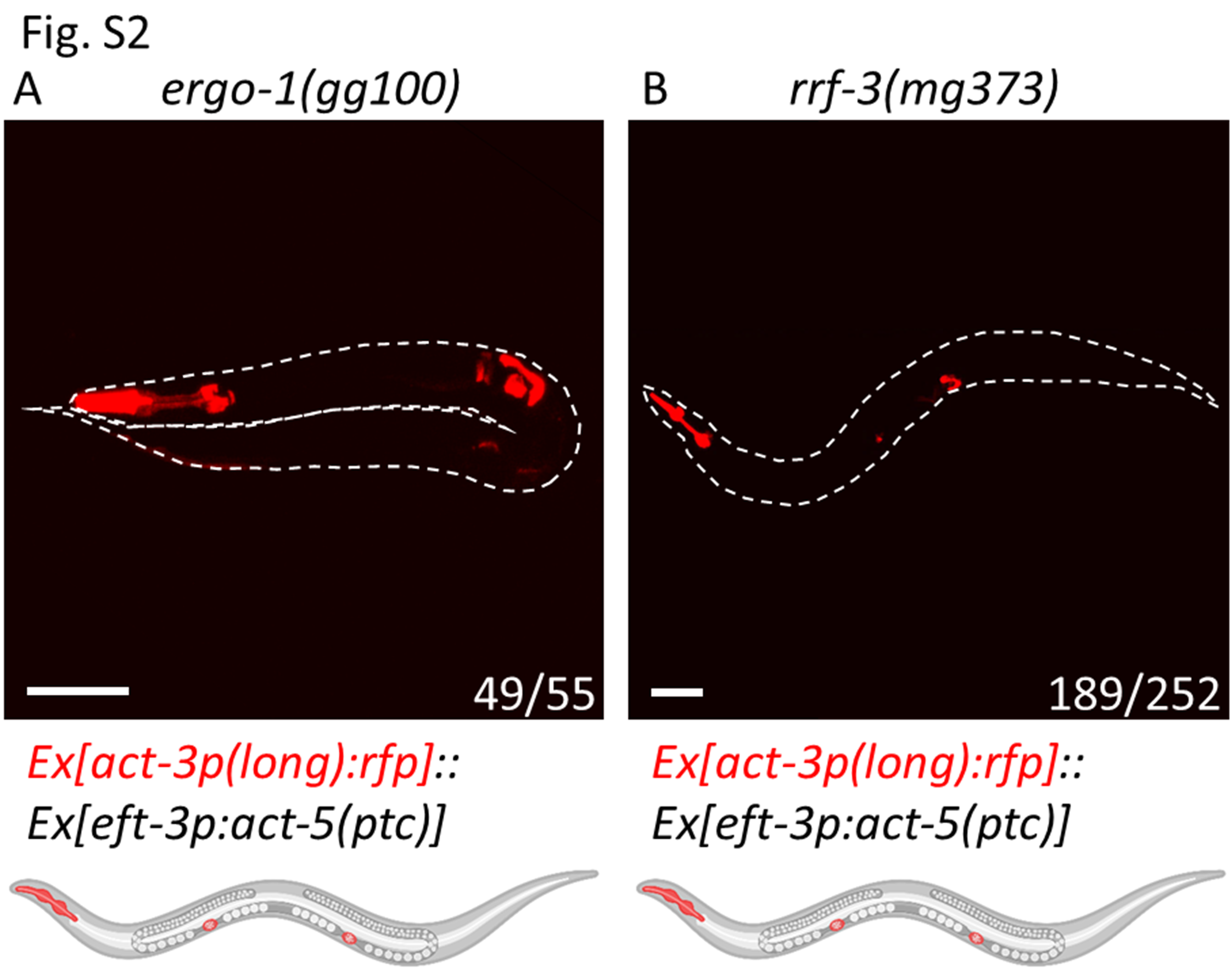

Supplement: S2 Fig — (A) Representative image and cartoon of an ergo-1 mutant animal containing both the act-3p:rfp reporter and act-5(ptc) overexpression constructs displaying reporter expression in the pharynx and spermatheca. (B) Representative image and cartoon of an rrf-3 mutant animal containing both the act-3p:rfp reporter and act-5(ptc) overexpression constructs displaying reporter expression in the pharynx and spermatheca. Worms outlined with a white dotted line; n = number of animals exhibiting the representative phenotype over the number of fluorescent animals screened. Cartoons were generated using Biorender.com (full license). Scale bars = 100 μm. (TIF) [file pgen.1010806.s002.tif]

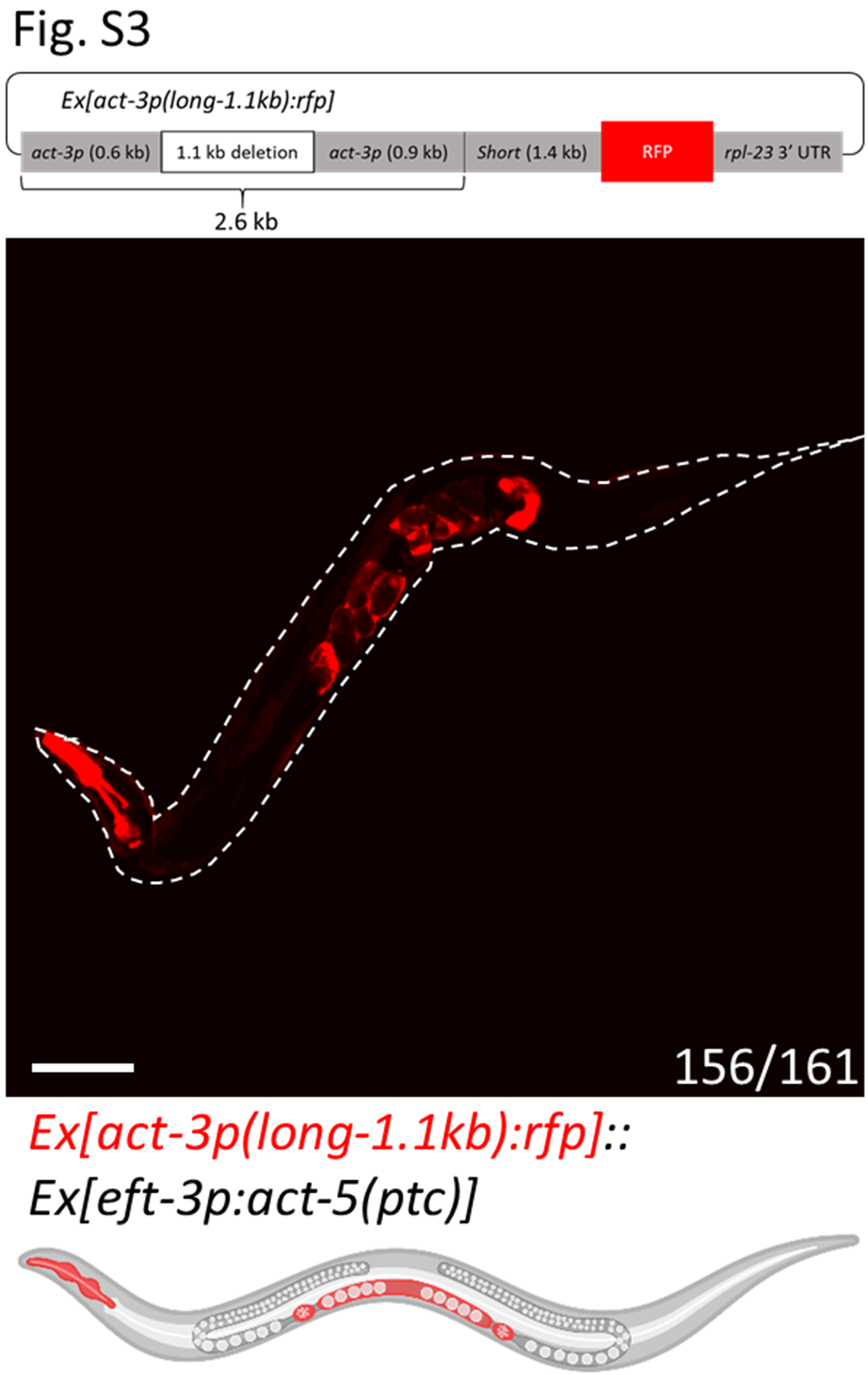

Supplement: S3 Fig — Diagram of the [act-3p(long-1.1kb):rfp] reporter construct. A 1.1 kb sequence was removed from the middle of the 2.6 kb region in the [act-3p(long):rfp] construct. Representative image and cartoon of an animal containing both the act-3p:rfp reporter and act-5(ptc) overexpression constructs displaying reporter expression in the pharynx, spermatheca, and uterus. Worms outlined with a white dotted line; n = number of animals exhibiting the representative phenotype over the number of fluorescent animals screened. Cartoons were generated using Biorender.com (full license). Scale bar = 100 μm. (TIF) [file pgen.1010806.s003.tif]

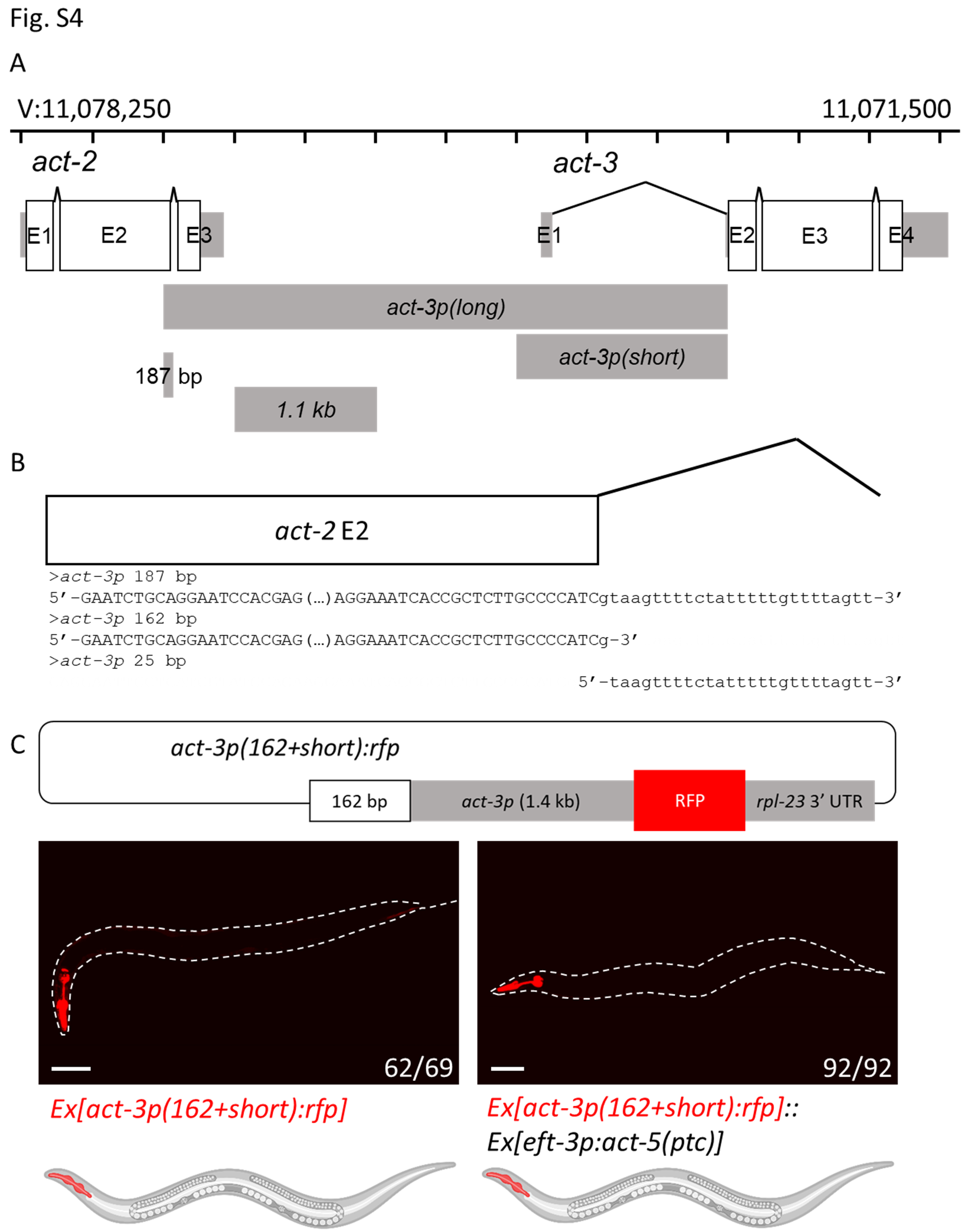

Supplement: S4 Fig — (A) Diagram of the act-2/act-3 locus (PRJNA13758:WBcel235) [16]. Exons (E) are displayed as UTRs (grey boxes) and coding sequence (white boxes), and introns are represented by bent lines. act-3p(long), act-3p(short), 1.1 kb deletion, and 187 bp sequences are also displayed as grey boxes. Tick marks represent 500 bp. (B) Diagram of the 5’ end of the [act-3p(long):rfp] reporter construct. The 5’ 187 bp element is located in the neighboring act-2 locus. The 187 bp element consists of the 3’ 161 bp of act-2 exon 2 (white box), and 26 bp of act-2 intron 2 (bent line). This element was further tested as the 5’ 162 bp segment, and the 3’ 25 bp segment. Sequences are listed below. (C) Diagram of the [act-3p(162+short):rfp] reporter construct. 162 bp from the 5’ end of the [act-3p(long):rfp] reporter construct were added to the 5’ end of the [act-3p(short):rfp] reporter construct. Representative image and cartoon of a control animal (left) displaying reporter expression in only the pharynx. Representative image and cartoon of an animal (right) containing both the [act-3p(162+short):rfp] reporter and act-5(ptc) overexpression constructs displaying reporter expression in only the pharynx. Worms outlined with a white dotted line; n = number of animals exhibiting the representative phenotype over the number of fluorescent animals screened. Cartoons were generated using Biorender.com (full license). Scale bar = 100 μm. (TIF) [file pgen.1010806.s004.tif]

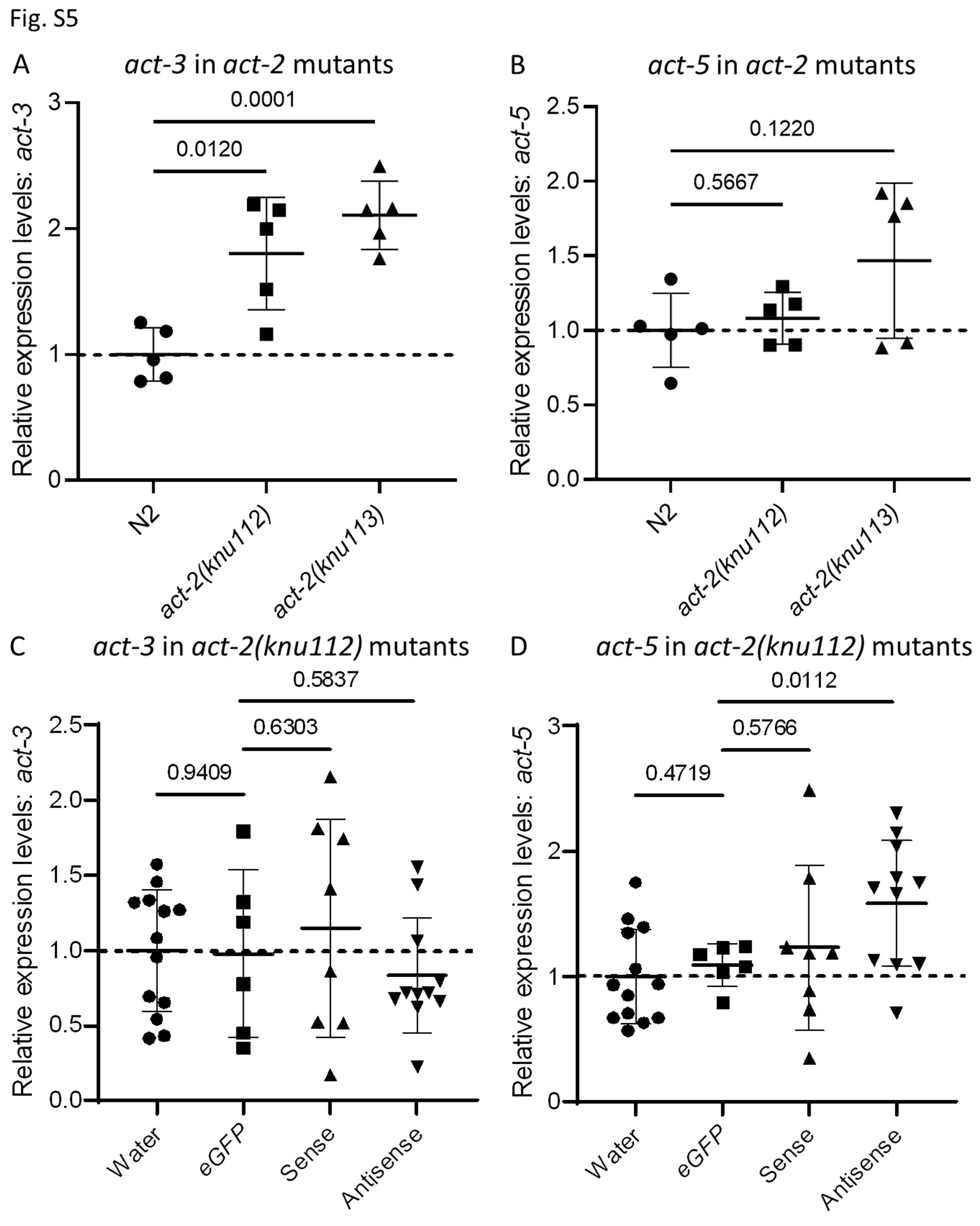

Supplement: S5 Fig — (A) Relative mRNA levels of act-3 in WT, act-2(knu112) mutant, and act-2(knu113) mutant animals. (B) Relative mRNA levels of act-5 in WT, act-2(knu112) mutant, and act-2(knu113) mutant animals. n ≥ 5 biologically independent samples. Data compared with WT controls. (C) Relative mRNA levels of act-3 in act-2(knu112) mutant animals injected with water, eGFP ssRNA, sense act-5 ssRNA, and antisense act-5 ssRNA. (D) Relative mRNA levels of act-5 in act-2(knu112) mutant animals injected with water, eGFP ssRNA, sense act-5 ssRNA, and antisense act-5 ssRNA. n ≥ 6 biologically independent samples. Data compared with water injected controls. Data are mean ± s.d., and a two-tailed Welch’s t-test was used to calculate P values. Ct values are listed in S4 Table. (TIF) [file pgen.1010806.s005.tif]
